# Diagnostic Concordance Between Clinical and Histopathologic Diagnoses of Oral Mucosal Lesions: A Retrospective Study

**DOI:** 10.7759/cureus.98026

**Published:** 2025-11-28

**Authors:** Patricia Hernandez-Rivera, Masoud Mirimoghaddam, Hollis Lai, Pallavi Parashar

**Affiliations:** 1 Faculty of Medicine and Dentistry, University of Alberta, Edmonton, CAN

**Keywords:** clinical diagnosis, concordance, descriptive analysis, histopathology, oral lesions

## Abstract

Introduction

Accurate diagnosis is essential for effective treatment and for determining the outcome of any pathologic condition. This study aimed to evaluate the concordance between the clinical and histopathologic diagnoses of biopsied soft-tissue specimens submitted to the Oral Pathology Biopsy Service at the University of Alberta over a 23-year period.

Methods

The clinical and histopathological diagnoses were coded using the Systematized Nomenclature of Medicine-Clinical Terms (SNOMED-CT). Subsequently, all the diagnoses were classified according to their pathogenesis. The outcome measurement was the percentage of absolute concordance, relative concordance, and discordance. Diagnostic agreement was evaluated using Cohen’s kappa; sensitivity, specificity, and positive and negative predictive values (PPV and NPV, respectively) were calculated. Additionally, the relationship between gender, age, and pathogenic-cluster concordance was tested using the Chi-square test or the Sample t-test.

Results

The anonymized database comprised 18,935 oral soft-tissue biopsies. The absolute concordance by SNOMED-CT codes was 47.8%, and by synonyms, 52.1%. The relative concordance was 76.2%, and the discordance was 23.8%. The accuracy of the clinical diagnosis in detecting oral potentially malignant disorders (OPMDs) was evaluated, yielding a sensitivity of 79.3%, a specificity of 97.9%, a PPV of 88.4%, and an NPV of 95.9%. Moreover, for malignant lesions, the clinical diagnosis demonstrated a sensitivity of 67.8%, a specificity of 98.5%, a PPV of 47.0%, and an NPV of 99.4%.

Conclusions

The results indicated that cluster concordance demonstrated substantial agreement. While clinical examination effectively identifies patients without malignancy or OPMD, it is not sufficiently sensitive to diagnose them. Therefore, histopathologic evaluation of biopsy specimens remains critical for achieving an accurate and definitive diagnosis.

## Introduction

The art of establishing a clinical or differential diagnosis requires knowledge, clinical expertise, and the ability to create mental pathways that lead to a definitive diagnosis. This diagnostic reasoning process involves integrating the patient's medical history, the history of the presenting complaint, and the observed clinical manifestations [1-5]. It is crucial to accurately differentiate malignant and oral potentially malignant disorders (OPMDs) from benign conditions, especially when different pathologies exhibit similar histopathologic features. This differentiation must be corroborated with the clinical impression to ensure an accurate diagnosis [3,6,7]. Histopathologic examination remains the gold standard, particularly for unresolved or clinically suspicious lesions [3,7]. Therefore, a biopsy is indicated when an oral pathologic lesion fails to improve within two weeks of eliminating irritational factors, in order to rule out an OPMD or other pathology of unknown etiology [2].

Several studies have evaluated the clinical-histopathologic concordance in oral pathology, with results ranging from 49.4% to 72% [3,6,8-13]. According to previous studies, the concordance rate is lower when researchers compare exact terminology between clinical and histopathological diagnoses [8,10,12]. Therefore, it is important to highlight that the methodologies used to classify pathologies vary across these studies, which may account for differences in reported concordance rates (Appendix Table 3) [3,6,8-13].

The present research aimed to evaluate the diagnostic accuracy of clinical impressions compared with histopathologic diagnoses of biopsy specimens submitted to the Oral Pathology Biopsy Service at the University of Alberta by licensed dentists over a 23-year period from 1985 to 2008. The outcome measurement was the percentage of absolute concordance, relative concordance, and discordance. As a secondary outcome, sensitivity, specificity, and positive and negative predictive values (PPV and NPV, respectively) were also analyzed. In addition, we aimed to apply a novel methodological approach to enhance the precision of this comparative analysis.

## Materials and methods

This retrospective study analyzed pathology reports from specimens submitted to the Oral Pathology Biopsy Service at the University of Alberta, Edmonton, Canada, between 1985 and 2008. The University of Alberta managed the Oral Pathology Biopsy Service until 2008; therefore, access to those reports was available during this period. Recent advancements in digitizing pathology reports allowed for the compilation of reports from this period [14].

The anonymized database contained biographic data from patients and diagnostic information. All reports with complete clinical and histopathologic diagnoses of oral soft-tissue biopsies were included. Ambiguous or nonspecific diagnoses that provided a descriptive term instead of a definitive diagnosis, for example, chronic inflammation, were excluded from the analysis. Likewise, intraosseous oral pathology, extraoral pathology, oropharyngeal pathologies, and cytology reports were excluded. Reports with more than two biopsies representing different pathologies from distinct sites and etiologies were separated and included as individual entries. Conversely, multiple incisional biopsies for the same pathology were counted as a single case.

Only the first listed clinical diagnosis was selected for comparison with the histopathologic diagnosis, as it represents the most likely diagnosis according to the clinician. The clinical and histopathologic diagnoses were refined to summarize the diagnosis in a single concept, using the current nomenclature and representing a pathology or condition without altering the concept or meaning stipulated by the clinician or the pathologist. Diagnoses with obsolete terminology were replaced with the currently accepted nomenclature; for example, “mucous retention cyst” was superseded by the current term “salivary duct cyst.” Hence, the words were extracted, prioritizing those representing malignancies, followed by OPMD and benign conditions.

All the diagnostic terms were assigned their corresponding numerical code in the Systematized Nomenclature of Medicine Clinical Terms (SNOMED-CT) online browser [15].

Several synonymous diagnostic terms exist that are assigned independent SNOMED-CT codes despite representing the same pathological entity (Appendix Table 4). To mitigate the potential reduction in concordance accuracy caused by this issue, a secondary synonym list was developed based on the textbook Oral and Maxillofacial Pathology by Neville et al., allowing conceptually equivalent conditions to be grouped under a single, unified code [16].

A classification system was created that was adapted from the categorization used by Soyele et al. [8] to categorize the pathologies based on their pathogenesis. This process yielded 21 distinct pathologic clusters, which were subsequently classified according to their prognosis as benign, OPMD, or malignant. The clustering framework was initially conducted by a former oral medicine graduate student, who categorized each condition based on the pathogenesis and prognosis. This classification was reviewed and validated by an oral pathologist, using oral pathology textbooks and consensus reports as references [16-21]. In the OPMD cluster, the pathologies included corresponded to those recognized in the World Health Organization consensus report [18].

**Figure 1:**
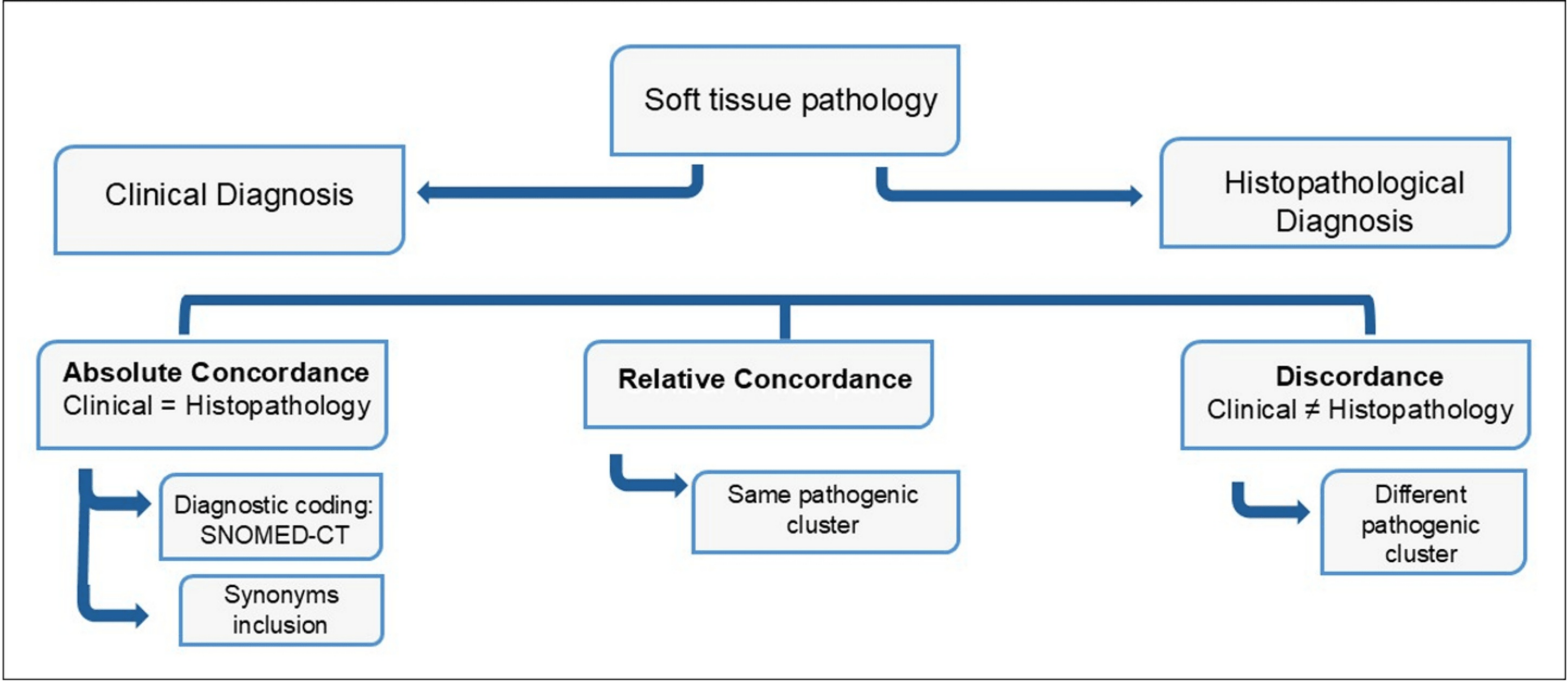
Flowchart analysis of the concordance between clinical and histopathological diagnosis SNOMED-CT: Systematized Nomenclature of Medicine-Clinical Terms

The concordance analysis between the clinical and histopathologic diagnoses was performed at three levels: a specific comparison utilized SNOMED-CT codes as the first level. Next, synonyms were integrated into the analysis. At this first level, “Absolute Concordance” was defined as identical SNOMED-CT codes, after considering the synonyms. Secondly, a less granular review compared all the diagnoses according to the pathogenic clusters to which they belonged. Therefore, “Relative Concordance” was determined when both diagnoses belonged to the same pathogenic cluster, and “Discordance” when they belonged to different clusters. All the discordant diagnoses were further analyzed, considering the prognostic implications. This analysis process is graphed in Figure 1.

All statistical analyses were completed using IBM SPSS Statistics for Windows, Version 29 (Released 2022; IBM Corp., Armonk, NY, USA). The demographic information was analyzed using descriptive statistics. The absolute concordance, relative concordance, and discordance were presented as percentages. Cohen’s Kappa was used to evaluate the concordance rate between clinical and histological accuracies for absolute and relative concordance. The level of agreement was determined as follows: <0.00, poor; 0.00-0.20, slight; 0.21-0.40, fair; 0.41-0.60, moderate; 0.61-0.80, substantial; and 0.81-1.00, almost perfect [22]. The statistical significance of age, gender, and pathological cluster concordance was calculated using a Chi-square test and a Sample t-test; a p-value less than 0.05 was considered significant. Moreover, the clinical diagnostic accuracy of detecting benign, OPMD, and malignant pathologies was analyzed using sensitivity, specificity, PPVs, and NPVs, respectively, as a multiclass analysis including these three categories.

## Results

All oral soft tissue biopsies received by the Oral Pathology Biopsy Service at the University of Alberta from 1985 through 2008 were considered for this study. A total of 18,935 cases fulfilled the inclusion criteria for the research analysis. The gender distribution was 9,955 (52.6%) females, 8,658 (45.7%) males (female:male ratio 1.15:1), and 322 (1.7%) of unknown gender. The age distribution included children (less than 14 years), 1,113 (5.8%); youths (15-24 years), 1,292 (6.8%); adults (25-64 years), 12,294 (65.0%); seniors (over 65 years), 3,533 (18.7%); and unknown age, 703 (3.7%). The mean age was 47.7 years, with a standard deviation of 20.2 and a standard error of 0.15. The skewness was 0.18, and the kurtosis was -0.14, demonstrating a slightly right-skewed and fairly symmetrical distribution with fewer extreme values.

**Figure 2:**
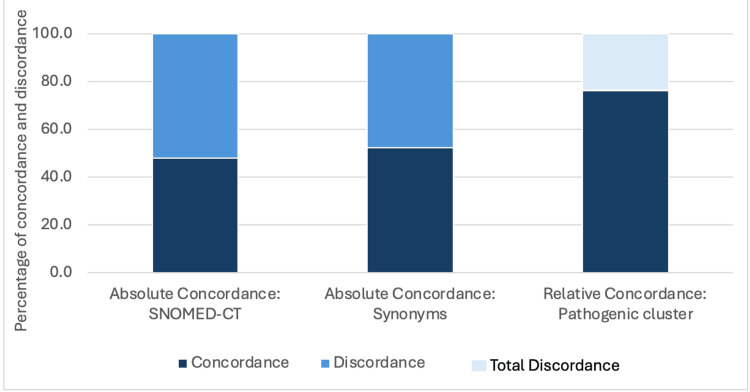
Concordance between clinical and histopathologic diagnoses SNOMED-CT: Systematized Nomenclature of Medicine-Clinical Terms

The absolute concordance between the clinical and histopathologic diagnoses, comparing the SNOMED-CT codes, was observed in 9,047 cases (47.8%), with a Cohen’s Kappa value of 0.41, demonstrating fair agreement. When the diagnostic synonyms were considered, the absolute concordance increased to 9,870 cases (52.1%). The relative concordance by pathogenic clusters was achieved in 14,427 cases (76.2%), with a Cohen’s Kappa value of 0.68, demonstrating substantial agreement. The discordance between clinical diagnoses and histopathologic findings was identified in 4,508 cases, representing 23.8% of the total cases analyzed. For benign conditions, the clinical diagnosis demonstrated a sensitivity of 97.0%, specificity of 82.3%, PPV of 95.9%, and NPV of 86.2%. Conversely, for malignant conditions, sensitivity was notably lower at 67.8%, while specificity remained high at 98.5%; the PPV was 47.0%, and the NPV was 99.4%. For OPMD, the clinical diagnosis yielded a sensitivity of 79.3%, specificity of 97.9%, PPV of 88.4%, and NPV of 95.9% (Figure 2 and Table 1).

**Table 1:** Sensitivity, specificity, PPV, and NPV for the clinical diagnosis of benign, OPMD, and malignant pathologies OPMD: Oral Potentially Malignant Disorder; PPV: Positive Predictive Value; NPV: Negative Predictive Value

Diagnosis	Sensitivity	Specificity	PPV	NPV
Benign	97.00%	82.30%	95.90%	86.20%
OPMD	79.30%	97.90%	88.40%	95.90%
Malignant	67.80%	98.50%	47.00%	99.40%

**Figure 3:**
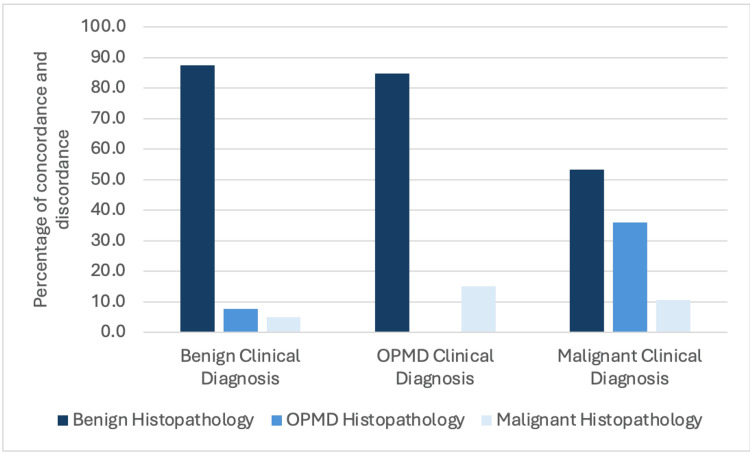
Analysis of discordant diagnoses by biological behavior OPMD: Oral Potentially Malignant Disorder

In the discordance group, when the clinical diagnosis was benign, 3,246 cases (87.4%) were misdiagnosed as a different benign pathology, while 284 cases (7.6%) were OPMD, and 184 cases (5.0%) were malignant. Conversely, when the clinical diagnosis was malignant, 71 cases (53.4%) were benign, 48 cases (36.1%) were OPMD, and 14 cases (10.5%) belonged to a different malignant cluster. When the clinician provided a tentative diagnosis of an OPMD, 561 cases (84.9%) were benign, and 100 cases (15.1%) were malignant (Figure 3). 

Concordance between clinical and histopathologic diagnosis according to the pathogenic clusters

The concordance between the clinical and histologic diagnoses was analyzed within each pathogenic cluster. The highest absolute concordance was observed in the benign virally-induced verrucopapillary cluster (74.5%), followed by the foreign body reaction cluster (67.3%) and the reactive lesions cluster (60.5%). The highest relative concordance was seen in the reactive pathologic cluster (82.6%), followed closely by the benign virally-induced verrucopapillary cluster (82.3%) and benign salivary gland pathologies (80.4%). In contrast, the lowest concordance was found in the salivary malignancy cluster (11.4%) and the benign soft tissue tumor cluster (16.9%) (Table 2).

**Table 2:** Concordance between clinical and histopathological diagnoses by etiopathologic cluster OPMD: Oral Potentially Malignant Disorder

Pathogenic Clusters	Absolute Concordance		Relative Concordance		Discordance		Total
	#	%	#	%	#	%	#
Reactive	5363	60.5	7326	82.6	1545	17.4	8871
Benign virally-induced verrucopapillary	1051	74.5	1161	82.3	249	17.7	1410
Benign salivary	955	53.7	1428	80.4	349	19.6	1777
OPMD	1083	33.9	2530	79.3	661	20.7	3191
Benign epithelial	108	37.2	218	75.2	72	24.8	290
Foreign body reactions	350	67.3	389	74.8	131	25.2	520
Lymphatic-hematologic	172	51	247	73.3	90	26.7	337
Epithelial malignancy	154	50.3	218	71.2	88	28.8	306
Immune mediated	20	37	38	70.4	16	29.6	54
Soft tissue-hematologic malignancy	14	50	15	53.6	13	46.4	28
Ulcerative-inflammatory	399	33.4	566	47.3	630	52.7	1196
Fungal	37	38.9	43	45.3	52	54.7	95
Developmental	59	18.9	133	42.6	179	57.4	312
Soft tissue counterpart of odontogenic or bone pathologies	41	41.4	42	42.4	57	57.6	99
Bacterial	2	25	2	25	6	75	8
Benign soft tissue	60	15.1	67	16.9	330	83.1	397
Salivary malignancy	2	5.7	4	11.4	31	88.6	35
Choristoma	0	0	0	0	4	100	4
Metastasis	0	0	0	0	1	100	1
Non-infectious granulomatous inflammation of systemic origin	0	0	0	0	2	100	2
Viral	0	0	0	0	2	100	2

Concordance between the clinical and histopathologic diagnoses according to gender

The concordance between the clinical and histopathologic diagnoses was also calculated according to gender. In the female group, the absolute concordance included 5,306 cases (53.3%); by pathogenic cluster, 7,635 cases (76.7%); and discordance was noted in 2,320 cases (23.3%).

For the male group, the absolute concordance included 4,379 cases (50.6%); by pathogenic cluster, 6,528 cases (75.4%); and discordance accounted for 2,130 cases (24.6%).

Finally, in the group with unknown gender, absolute concordance was observed in 185 cases (57.5%); relative concordance in 264 cases (82.0%); and discordance in 58 cases (18.0%). The relationship between cluster concordance and gender was analyzed using a Pearson Chi-square, which demonstrated a statistically significant association between the two variables (p < 0.006) with a Phi value of 0.023, indicating a weak association.

Concordance between the clinical and histopathologic diagnoses according to age groups

The diagnostic concordance by age group was as follows: in the children group, absolute concordance was observed in 557 cases (50.0%); relative concordance by pathogenic clusters in 815 cases (73.2%); and discordance in 298 cases (26.8%). In the youth group, absolute concordance was observed in 688 cases (53.3%); relative concordance by pathogenic clusters in 978 cases (75.7%); and discordance in 314 cases (24.3%). In the adult group, absolute concordance was observed in 6,560 cases (53.4%); relative concordance by pathogenic clusters in 9,478 cases (77.1%); and discordance in 2,816 cases (22.9%). In the senior group, absolute concordance was observed in 1,703 cases (48.2%); relative concordance by pathogenic clusters in 2,623 cases (74.2%); and discordance in 910 cases (25.8%). Finally, in the unknown age group, absolute concordance was observed in 362 cases (51.5%); relative concordance by pathogenic clusters in 533 cases (75.8%); and discordance in 170 cases (24.2%). The relationship between cluster concordance and age was assessed using a Sample t-test, which showed no significant difference (p = 0.241).

## Discussion

This retrospective study evaluated the diagnostic concordance between the clinical impressions and the histopathologic diagnoses of 18,935 biopsy specimens submitted to the Oral Pathology Biopsy Service at the University of Alberta by licensed dentists between 1985 and 2008. This research utilized an extensive database, which significantly exceeds the combined sample sizes of all referenced studies, thereby providing unequivocal support for the validity of our findings. Another important feature of this study is the development of a novel methodological approach to enhance the precision of this comparative analysis [14].

This study determined an absolute concordance of 47.8%-52.1%, a relative concordance of 76.2%, and a discordance rate of 23.8%. The accuracy of the clinical diagnosis in detecting OPMD was evaluated, yielding a sensitivity of 79.3%, a specificity of 97.9%, a PPV of 88.4%, and an NPV of 95.9%. Moreover, for malignant lesions, the clinical diagnosis demonstrated a sensitivity of 67.8%, a specificity of 98.5%, a PPV of 47.0%, and an NPV of 99.4%. These results demonstrate that histopathology is an essential assessment to provide a definitive diagnosis, which will dictate further management requirements.

It is important to consider that certain clinical and histopathologic terms may describe the same entity; however, the terminology used in clinical diagnoses does not always align with that used in histopathologic diagnoses, and vice versa. One example of this dilemma is leukoplakia, a diagnosis defined by the World Health Organization as “a predominantly white plaque of questionable risk having excluded other known diseases or disorders that carry no increased risk for cancer” [18]. Another pertinent histologic term that has evolved over time is hyperkeratosis, including non-reactive hyperkeratosis. The latter represents architectural evidence of dysplasia when the thickness of the keratin layer is half or more of that of an atrophic epithelium, characterized by corrugation of the surface and loss of rete ridges in the scenario of absent cytological dysplasia [17,23].

This evolving understanding of terminology creates challenges when analyzing clinical and histopathologic diagnoses in oral pathology. Several authors have used a redefined concordance framework to overcome this terminology dilemma [10,12,13,19]. In the present study, we addressed this issue by creating a categorization of soft tissue pathologies, allowing for a comprehensive analysis based on pathogenesis and thereby providing a more consistent and biologically informed framework for diagnostic comparison. It is also important to emphasize that there is no universal classification of oral soft tissue pathologies and conditions [14]. Oral pathology textbooks group these conditions differently to facilitate the learning process [1,16,17]. As such, diagnostic concordance studies may yield differing results depending on the oral pathology classification used. For this reason, our research sought to overcome this limitation by employing a pathogenesis-based classification, enabling a more accurate comparison of clinical and histopathologic diagnoses. Moreover, this methodology can highlight the pathologic clusters in which clinicians commonly misdiagnose, thereby guiding further educational efforts and policies to improve patient management outcomes. 

Our study yielded an absolute concordance of 47.8%-52.1%, which is on the lower end of the range reported in the literature (52.6%-72.2%) [3,6,8-13]. This discrepancy may be attributed, in part, to differences in the methodology used to compare diagnostic terminology. Notably, our study employed the SNOMED-CT coding system, which offers a highly detailed and granular nomenclature [14]. This coding system has been proven to represent oral pathology terminology more precisely than other coding systems, including the International Classification of Diseases, providing the advantage of more granular data without losing information during the codification process [24,25]. In contrast, many previous studies did not clearly specify the criteria used for terminology selection or coding, potentially inflating concordance rates due to broader or less standardized categorizations. The variability in classification systems across studies further complicates inter-study comparisons. Mendez et al. also acknowledged this limitation, noting the difficulty in comparing their findings with those reported in the literature [13]. These methodological inconsistencies reinforce the importance of standardized diagnostic terminology, frameworks, and coding systems in future studies. 

A uniquely consistent feature across several studies is the diagnostic concordance within the reactive lesions group, with reported concordance rates ranging between 60.6% and 67.6% [6,8,12]. These findings are comparable to those in the present research, which demonstrated an absolute concordance rate of 61.1% and a relative concordance of 82.1% when grouped by pathogenic clusters.

In the discordant cases, where the diagnoses have different underlying causes, histopathologic examination often revealed a benign condition. However, a clinically significant subset of these discordant cases warrants particular attention, as some were ultimately diagnosed as OPMDs or malignancies, which would require a fundamentally different management approach. This underscores the critical importance of histopathologic assessment, particularly in cases where there is clinical suspicion of an OPMD or malignancy, as emphasized by the American Academy of Oral and Maxillofacial Pathology [26].

The analysis of concordance by gender in the present study showed higher concordance in females, with a statistically significant association between these variables, though the strength was weak. Soyele et al. and Forman et al. observed significantly higher concordance in the female group [8,11]. In contrast, Saravani et al. and Farzinnia et al. did not find any significant gender-based differences in diagnostic concordance [3,6].

The concordance according to age groups in the present study was highest in the adult group, although no statistically significant difference was found between age and cluster-based concordance. This finding aligns with those of Farzinnia et al. and Saravani et al., who also reported no significant correlation between age and diagnostic agreement [3,6]. Conversely, Soyele et al. reported the highest concordance in patients in their seventh decade of life, corresponding to the senior age group [8].

The sensitivity of clinical diagnoses for detecting OPMDs and malignancies was relatively low. This underscores the need for enhanced educational strategies aimed at improving clinicians’ ability to recognize these conditions and reinforces the critical role of histopathologic examination in achieving diagnostic accuracy and precision [27-29]. In contrast, the specificity for identifying true negative cases remains high, indicating that clinicians were effective in detecting patients who did not have OPMD or malignant conditions. As a result, clinical examinations minimize false positive cases, reducing the risk of overtreating patients who do not need treatment and alleviating any potential psychological implications associated with unnecessary interventions.

According to the meta-analysis by Walsh et al., the sensitivity for detecting OPMD and malignant conditions ranges from 0.5 to 1.0, and the specificity ranges from 0.9 to 1.0 [30]. Although the sensitivity and specificity reported in the present study fall within this published range, the findings highlight the continued need for ongoing education. As diagnostic concepts in OPMD and malignant disorders evolve, targeted training is essential to enhance clinical recognition and reduce the risk of diagnostic errors.

A key consideration is that studies from a specific medical facility have the advantage of being based on a captive population, facilitating logistics. However, they do not accurately represent the “real world” due to selection bias. This bias arises from the fact that patients are referred for assessment of a pathology or a suspected condition; thus, this kind of research does not contemplate the healthy portion of the population [31]. Even though the information is essential for understanding the epidemiological features of intraoral pathology, a critical difference between these investigations is that population-based studies focus on clinical diagnoses, while medical facility-based studies are primarily centered on histopathological diagnoses [32].

One recognized limitation of this study is the exclusion of certain reports that lacked complete data, a common issue in retrospective research. Another limitation lies in the changing understanding of certain conditions over time, particularly leukoplakia, which may affect diagnostic interpretation. To address this, we attempted to differentiate reactive from non-reactive hyperkeratosis in the analysis. Likewise, the intercoder agreement was not calculated.

It is relevant to highlight that the sensitivity of the clinical diagnosis of OPMD and malignancies is lower, requiring educational strategies to improve the diagnostic skills for these lesions, update clinical terminology, and corroborate the importance of histopathological diagnosis for an accurate assessment and understanding of the subsequent management required [27-29]. 

## Conclusions

In conclusion, the present research benefits from a substantial population size, facilitating a uniquely in-depth and clinically relevant analysis. Likewise, codifying diagnoses using SNOMED-CT and subsequently clustering by pathogenesis and prognosis offers a reliable method for determining the concordance between clinical and histopathologic diagnoses. This classification system helps address diagnostic discrepancies that may arise when clinicians and pathologists assign different diagnoses.

It is essential to note that the findings highlight a need to improve diagnostic acumen among licensed dental professionals - including general and specialized dental practitioners - particularly in recognizing OPMDs and malignancies. Ultimately, histopathologic evaluation is vital to secure an accurate diagnosis and guide appropriate treatment.

## Appendices

**Table 3:** Summarized studies evaluating the clinical-histopathologic concordance in oral pathology

Authors study	Country		Study period	Population size	Methodology	Results (Concordance %)
Soyele et al. [8]	Nigeria	2008-2017		592	Categories: Malignant and benign, the latter subdivided into: Reactive-hyperplastic lesions, cystic lesions, pulp and periapical lesions, giant cell lesions, fibro-osseous lesions, odontogenic tumors, epithelial tumors, mesenchymal tumors, salivary gland diseases, hemato-lymphoid neoplasms, inflammatory-microbial diseases, ulcerative lesions, normal tissue, and miscellaneous. Analysis: Concordance: Clinical and histopathological diagnosis exact.	Concordance 54.6%. Highest concordance in fibro-osseous lesions 65.6% and epithelial tumors 66.1%. Kappa coefficient: 0.5 (good).
Kondori et al. [9]	USA	2009-2010		976	No further classification. Comparison between general dentists and different specialties.	Overall concordance: 57%. General dentists: 54.1%. Maxillofacial surgeons: 57.2%. Endodontists: 57.8%. Periodontists: 58.8%.
Patel et al. [10]	New Zealand	2002-2006		3143	Categories: oral mucosal, gingival-periodontal, salivary, and miscellaneous. Re-categorized: Malignant, premalignant, benign, and no diagnosis, when no clinical diagnosis. Analysis: Concordant diagnosis: same clinical and histological (word by word). Concordant redefined example: Leukoplakia and epithelial hyperkeratosis with mild dysplasia. Discordance: different diagnoses. No clinical discordance.	Total concordance: 50.6% and 63.21% when the reports without a clinical diagnosis were deleted. The specialist’s total concordance was 51%, redefined to 62.7%. General dental practitioners 49.4% and redefined 66.7%.
Farzinnia et al. [6]	Iran	Jan 2006- Dec 2018		3001	Categories: 1. Ulcerative, vesicular and bullous, 2. Red and white, 3. Pigmented lesions, 4. Bone lesions. 5. Exophytic. Histopathology criteria, according to Neville. Analysis: Concordance: Similar diagnosis using both techniques.	Clinical and histopathological consistent 72.2%. The red and white lesions had the highest rate of concordance (86.1%), while the pigmented lesions had the lowest concordance (47.1%).
Saravani et al. [3]	Iran	April 1999- September 2015		631	Categories: Neoplastic and non-neoplastic. Clinical diagnosis according to priority: first, second, and third.	Diagnostic compatibility: 70.1%. First diagnosis: 87.2%. Second diagnosis: 10.6%. Third diagnosis: 2.1%.
Forman et al. [11]	United States of America	2005-2013		1003	Categories: 1. Premalignant or malignant 2. Benign. Analysis: Concordance: Same diagnosis, and then according to the groups. Accuracy: Both belonged to the same category, even if histology differed.	The concordance by specific diagnosis: 61%, according to category 94.4%.
Poudel et al. [12]	Nepal	Jan 2016-Dec 2017		237	Categories: Non-neoplastic-reactive, potentially malignant oral lesions, benign, malignant, non-odontogenic cysts & pseudocysts, odontogenic cysts, odontogenic tumors, and others. Analysis: Total concordance: Total agreement between clinical and histopathological. Concordance with the histopathological diagnosis but after refinement of clinical diagnosis.	Total concordance: 56.3%. Concordance with the histopathological diagnosis but after refinement of clinical diagnosis: 67.9%. Discordance: 23.6%. No clinical diagnosis: 8.4%. The maximum agreement was among benign, 73.17%.
Mendez et al. [13]	Brazil	1995-2004		5368	Categories: Inflammatory, benign, malignant, other, and then subclassified. Analysis: Clinical and histopathologic diagnosis from the same diagnostic group.	Agreement by groups: Periapical lesions 92.6%, Potentially malignant 90.1%, Non-neoplastic proliferative disorders 89.3%.

**Table 4:** Classification of soft tissue conditions

I. Bacterial cluster				
Pathology/Condition	SNOMED-CTcode	Synonyms	Prognosis	-
Bacterial infection	312128007		Benign	
Actinomycosis	23014006		Benign	
Syphilis	235062007		Benign	
Tuberculosis	235067001		Benign	
II. Benign epithelial cluster				
Pathology/Condition	SNOMED-CTcode	Synonyms	Prognosis	
Melanotic macule	3449001		Benign	
Melanin pigmentation	235038002	Oral pigmentation	Benign	
Oral pigmentation	249405005		Benign	
Mucosal melanosis	724847001		Benign	
Oral nevus	140051000119109	Blue nevus/intramucosal nevus/junctional nevus/compound nevus	Benign	
Blue nevus	63166000		Benign	
Intramucosal nevus	449767002		Benign	
Junctional nevus	30494009		Benign	
Compound nevus	49409001		Benign	
Verruciform xanthoma	708013001		Benign	
Keratoacanthoma	254662007		Benign	
III. Benign Salivary cluster				
Pathology/Condition	SNOMED-CTcode	Synonyms	Prognosis	
Salivary adenoma	1187379001		Benign	
Benign salivary gland neoplasia	34433006		Benign	
Adenomatoid hyperplasia of minor salivary gland	109763004		Benign	
Basal cell adenoma	27230006		Benign	
Canalicular adenoma	128641003		Benign	
Warthin tumor	422470007		Benign	
Intraductal papilloma	5244003		Benign	
Benign lymphoepithelial lesion	45517002		Benign	
Mucocele	69825009		Benign	
Oral ranula	14919007		Benign	
Mucous retention cyst	1260281008		Benign	
Necrotizing sialometaplasia	109769000		Benign	
Monomorphic adenoma	77653004		Benign	
Pleomorphic adenoma	8360001		Benign	
Blocked salivary gland	23512004		Benign	
Benign salivary gland neoplasia	255154009		Benign	
Salivary gland hyperplasia	698070009		Benign	
Sialadenitis	42982001		Benign	
Sialolith	28826002		Benign	
Sjogren's (lip biopsies)	83901003		Benign	
IV. Benign soft tissue cluster				
Pathology/Condition	SNOMED-CTcode	Synonyms	Prognosis	
Angioleiomyoma	86959002		Benign	
Amyloidosis	56871000		Benign	
Benign mesenchymal tumor	722691002		Benign	
Oral focal mucinosis	109786006		Benign	
Giant cell fibroma	109790008		Benign	
Granular cell tumor	404035005		Benign	
Gingival fibromatosis	58569000		Benign	
Fibrous histiocytoma	25889007		Benign	
Oral lipoma	404061009		Benign	
Fibrolipoma	2710003	Oral lipoma	Benign	
Neurofibroma	687111000119102		Benign	
Neurofibromatosis	81669005		Benign	
Schwannoma	985004		Benign	
Pigmented neuroectodermal tumor of infancy	404042005		Benign	
Leiomyoma	404042005		Benign	
V. Benign virally induced verruco-papillary cluster				
Pathology/Condition	SNOMED-CT code	Synonyms	Prognosis	
Condyloma	733132008		Benign	
Hecks disease	6121001		Benign	
Squamous papilloma	698182002	Papilloma	Benign	
Oral papilloma	402908003		Benign	
Papillomatosis	88172005		Benign	
Verruca vulgaris	57019003		Benign	
VI. Choristoma cluster				
Pathology/Condition	SNOMED-CT code	Synonyms	Prognosis	
Osseous choristoma	404075002		Benign	
VII. Developmental origin and variations of anatomy cluster				
Pathology/Condition	SNOMED-CT code	Synonyms	Prognosis	
Dermoid cyst	90365003		Benign	
Developmental cyst	12143007		Benign	
Epidermoid cyst	196548000		Benign	
Lymphoepithelial cyst	67045005		Benign	
Thyroglossal duct cyst	699000000		Benign	
Nasolabial cyst	90516007		Benign	
White sponge nevus	389203001		Benign	
Ankyloglossia	67787004		Benign	
Fat pad	5398002		Benign	
Frenal tag	698842006		Benign	
Hyperplastic tongue papilla	6971002		Benign	
Hyperplastic fungiform papilla	249384007	Hyperplastic tongue papilla	Benign	
Hyperplastic filiform papilla	255225007	Hyperplastic tongue papilla	Benign	
Hyperplastic circumvallate papillae	249385008	Hyperplastic tongue papilla	Benign	
Fordyce granules	50584008		Benign	
Sebaceous gland hyperplasia	238748009		Benign	
Sebaceous cyst	417992006			
Geographic tongue	59032001		Benign	
Black hairy tongue	81934005		Benign	
Leukoedema	67795000		Benign	
Linea alba	709495007		Benign	
Lymph nodes	30746006		Benign	
Lymphoid hyperplasia	43961000		Benign	
Physiological pigmentation	403218008		Benign	
Normal	162010006		Benign	
Operculum	10602003		Benign	
Retrocuspid papilla	110601005		Benign	
Double lip	699762000		Benign	
Graft tissue	260667007		Benign	
VIII. Epithelial malignant cluster				
Pathology/Condition	SNOMED-CT code	Synonyms	Prognosis	
Adenosquamous carcinoma	403902008		Malignant	
Squamous cell carcinoma	307502000		Malignant	
Spindle cell carcinoma	65692009		Malignant	
Verrucous carcinoma	403889000		Malignant	
Melanoma	403926005		Malignant	
Oral carcinoma	722688002		Malignant	
Basal cell carcinoma (clinical diagnosis)	1338007		Malignant	
IX. Foreign body reaction cluster				
Pathology/Condition	SNOMED-CT code	Synonyms	Prognosis	
Amalgam tattoo	109789004	Argyria	Benign	
Argyria	77783001		Benign	
Unspecific foreign body	14380007		Benign	
Foreign body granuloma	37058002	Suture granuloma	Benign	
Suture granuloma	66962008		Benign	
Heavy metal pigmentation	30771009		Benign	
Foreign body pigmentation	235045002		Benign	
X. Fungal cluster				
Pathology/Condition	SNOMED-CT code	Synonyms	Prognosis	
Oral candidiasis	79740000	Hyperplastic candidiasis/hypertrophic candidiasis/hypertrophic candidiasis/ chronic hyperplastic candidiasis	Benign	
Hyperplastic candidiasis	110277001		Benign	
Hypertrophic candidiasis	402997002		Benign	
Chronic hyperplastic candidiasis	235072005		Benign	
Fungal infection	3218000		Benign	
Denture stomatitis	69254008		Benign	
Median rhomboid glossitis	707318002		Benign	
XI. Immune-mediated cluster				
Pathology/Condition	SNOMED-CT code	Synonyms	Prognosis	
Autoimmune disease	85828009		Benign	
Erythema multiforme	36715001		Benign	
Desquamative gingivitis	22208002		Benign	
Mucous membrane pemphigoid	402441007		Benign	
Pemphigus vulgaris	49420001		Benign	
Scleroderma	267874003		Benign	
Psoriasis	9014002		Benign	
Behcet	310701003		Benign	
XII. Lymphatic-Hematological cluster				
Pathology/Condition	SNOMED-CT code	Synonyms	Prognosis	
Angiomatosis	14350002		Benign	
Caliber persistent artery	711100005		Benign	
Clot	75753009		Benign	
Endarteritis	33806008		Benign	
Hereditary hemorrhagic telangiectasia	21877004		Benign	
Oral hemangioma	403963001	Cavernous hemangioma	Benign	
Cavernous hemangioma	33377007		Benign	
Hematoma	262648004		Benign	
Petechia	50091001		Benign	
Langerhans cell histiocytosis	8090002		Benign	
Lymphangioma	238803001		Benign	
Phlebolith	37876005		Benign	
Spider angioma	195382003		Benign	
Thrombus	396339007		Benign	
Varicosity	702792005	Vascular anomaly/ arteriovenous malformation/angiopathy	Benign	
Vascular anomaly	783806000		Benign	
Arteriovenous malformation	24551003		Benign	
Angiopathy	27550009		Benign	
Intravascular papillary endothelial hyperplasia	238770007		Benign	
Thrombocytopenic purpura	302873008		Benign	
XIII. Metastasis cluster				
Pathology/Condition	SNOMED-CT code	Synonyms	Prognosis	
Metastasis	404094007		Malignant	
XIV. Oral Potentially Malignant Disorder cluster				
Pathology/Condition	SNOMED-CT code	Synonyms	Name cluster	Prognosis
Carcinoma in situ	92660005		OPMD	OPMD
Atypia	50673007		OPMD	OPMD
Dysplasia	61313004		OPMD	OPMD
Erythroplakia	69299000		OPMD	OPMD
Non-reactive hyperkeratosis	249409004		OPMD	OPMD
Graft versus host disease	402362009		OPMD	OPMD
Verrucous hyperplasia	109785005		OPMD	OPMD
Leukoplakia	414603003		OPMD	OPMD
Verrucous leukoplakia	235031008		OPMD	OPMD
Erythroleukoplakia	698199001		OPMD	OPMD
Lichenoid reaction	235050008	Lichenoid lesion	OPMD	OPMD
Lichenoid mucositis	699290002		OPMD	OPMD
Oral lichen planus	4776004		OPMD	OPMD
Erosive lichen planus	238662007	Oral lichen planus	OPMD	OPMD
Lupus	707301001		OPMD	OPMD
Oral submucous fibrosis	32883009		OPMD	OPMD
Premalignancy	1269006002		OPMD	OPMD
XV. Non infectious Granulomatous Inflammation of Systemic origin cluster				
Pathology/Condition	SNOMED-CT code	Synonyms	Prognosis	-
Crohns disease	196578009		Benign	
Wegener granulomatosis	195353004		Benign	
Sarcoidosis	707238003		Benign	
XVI. Reactive pathologies cluster				
Pathology/Condition	SNOMED-CT code	Synonyms	Prognosis	
Angina bullosa haemorrhagica	235025005		Benign	
Dystrophic calcification	60963005		Benign	
Inflammatory fibrous hyperplasia	1137562007	Epulis/leaf fibroma	Benign	
Epulis	45676007		Benign	
Leaf fibroma	419585004		Benign	
Irritation fibroma	1.68948E+16	Fibroepithelial polyp/fibrous nodule	Benign	
Fibroepithelial polyp	1141622007		Benign	
Fibrous nodule	11854003		Benign	
Fibrous tissue	34433006		Benign	
Frictional keratosis	235034000		Benign	
Drug induced gingival hyperplasia	93434009		Benign	
Hyperplastic tissue	76197007		Benign	
Focal epithelial hyperplasia	6121001	Epithelial hyperplasia	Benign	
Epithelial hyperplasia	31390008		Benign	
Hypertrophic scar	19843006		Benign	
Inflammatory papillary hyperplasia	41349008		Benign	
Oral neuroma	1163436007	Traumatic Neuroma	Benign	
Pyogenic granuloma	17372009		Benign	
Morsicatio buccarum	59901004		Benign	
Nicotine stomatitis	89013002		Benign	
Peripheral giant cell granuloma	89722009		Benign	
Peripheral ossifying fibroma	109788007		Benign	
Pregnancy tumor	235003004	Pyogenic granuloma	Benign	
Epithelial polyp	41329004		Benign	
Postinflammatory pigmentation	95348005		Benign	
Reactive tissue	402870005		Benign	
Hyperkeratosis (Reactive)	739032003		Benign	
Smoker’s melanosis	5661000124106		Benign	
Smoker’s keratosis	235033006		Benign	
Smokeless tobacco keratosis	95269005		Benign	
Scar tissue	12402003		Benign	
Traumatic neuroma	230650009	Oral neuroma	Benign	
Traumatic injury	417746004		Benign	
Pseudoepitheliomatous hyperplasia	254665009		Benign	
XVII. Salivary malignancy cluster				
Pathology/Condition	SNOMED-CT code	Synonyms	Prognosis	
Mucoepidermoid carcinoma	423708008		Malignant	
Acinic cell carcinoma	45410002		Malignant	
Polymorphous adenocarcinoma	128702009		Malignant	
Adenocarcinoma	443961001		Malignant	
Adenoid cystic carcinoma	422833009		Malignant	
Clear cell adenocarcinoma	30546008		Malignant	
Salivary malignancy	255072001		Malignant	
XVIII. Soft tissue counterpart of odontogenic and bone pathology cluster				
Pathology/Condition	SNOMED-CT code	Synonyms	Prognosis	
Eruption cyst	42323001		Benign	
Gingival cyst	58271001		Benign	
Peripheral ameloblastoma	278404007		Benign	
Peripheral odontogenic fibroma	75914009		Benign	
Peripheral odontogenic tumor	276968006		Benign	
XIX. Soft tissue and hematological malignancies cluster				
Pathology/Condition	SNOMED-CT code	Synonyms	Prognosis	
Kaposi sarcoma	1217617009		Malignant	
Lymphoma	118600007		Malignant	
Leiomyosarcoma	443719001		Malignant	
Leukemia	93143009		Malignant	
Plasmacytoma	188718006		Malignant	
Sarcoma	424413001		Malignant	
XX. Ulcerative-inflammatory cluster				
Pathology/Condition	SNOMED-CT code	Synonyms	Prognosis	
Chronic abscess	81546003	Gingival periodontal nonspecific inflammation	Benign	
Chronic infection	275393007	Gingival periodontal nonspecific inflammation	Benign	
Acute inflammation	4532008	Gingival periodontal nonspecific inflammation	Benign	
Periodontal abscess	83412009	Gingival periodontal nonspecific inflammation	Benign	
Allergic reaction	402251006		Benign	
Aphthous	426965005		Benign	
Chemical burn	1.21081E+16	Traumatic ulcer	Benign	
Chronic inflammation	20369000	Gingival periodontal nonspecific inflammation	Benign	
Non-specific inflammation	61170000	Gingival periodontal nonspecific inflammation	Benign	
Oral fistula	20674003	Fistula	Benign	
Oroantral fistula	109675004	Fistula	Benign	
Oral granuloma	45647009		Benign	
Granulation tissue	61363009	Gingival periodontal nonspecific inflammation	Benign	
Gingival hyperplasia	54711002	Gingival periodontal nonspecific inflammation	Benign	
Unspecific gingivitis	66383009	Gingival periodontal nonspecific inflammation	Benign	
Hyperplastic gingivitis	84161008	Gingival periodontal nonspecific inflammation	Benign	
Glossitis	45534005		Benign	
Mucositis	95361005	Gingival periodontal nonspecific inflammation	Benign	
Necrosis	6574001		Benign	
Acute necrotizing ulcerative gingivitis	707792000		Benign	
Foliate papillitis	710003004		Benign	
Parulis	109610001	Fistula	Benign	
Pericoronitis	22240003	Gingival periodontal nonspecific inflammation	Benign	
Periodontitis	41565005	Gingival periodontal nonspecific inflammation	Benign	
Plasma cell gingivitis	234992005		Benign	
Thermal burn	403445007	Traumatic ulcer	Benign	
Traumatic ulcer	403444006		Benign	
Traumatic ulcer granuloma	403455006		Benign	
Non-specific ulcer	26284000	Traumatic ulcer	Benign	
Lymphocytic infiltrate	54727009		Benign	
Nodular fasciitis	400138001		Benign	
Periimplantitis	699422003		Benign	
XXI. Viral cluster				
Pathology/Condition	SNOMED-CT code	Synonyms	Prognosis	
Viral infection	34014006		Benign	
Herpes simplex	235058001		Benign	
Herpes zoster	235059009		Benign	
Hairy leukoplakia	414952002		Benign	
